# Correlation of Serum BACE1 With Emergence Delirium in Postoperative Patients: A Preliminary Study

**DOI:** 10.3389/fnagi.2020.555594

**Published:** 2020-10-28

**Authors:** Chunyan Ye, Yanrong Zhang, Sumei Luo, Yanan Cao, Feng Gao, E. Wang

**Affiliations:** ^1^Department of Anesthesiology, Xiangya Hospital Central South University, Changsha, China; ^2^Institute on Aging and Brain Disorders, The First Affiliated Hospital of USTC, Division of Life Sciences and Medicine, University of Science and Technology of China, Hefei, China; ^3^National Clinical Research Center for Geriatric Disorders (Xiangya Hospital), Changsha, China

**Keywords:** general anesthesia, emergence, delirium, biomarker, BACE1

## Abstract

**Background**: The mechanism underlying delirium, a common acute fluctuating mental state, may be related to the activation of a neuroinflammatory response. In this study, we attempted to investigate whether plasma inflammatory response markers, vascular and cerebrovascular injury-related markers, and neurodegeneration-associated markers were associated with emergence delirium (ED).

**Methods**: Patients aged 50 years or above who underwent elective laparoscopic surgery under general anesthesia were included in this study. Delirium was assessed postoperatively with the Richmond Agitation Sedation Scale (RASS) and the Confusion Assessment Method for the Intensive Care Unit (CAM-ICU) scale. Plasma samples were collected from ED patients and non-ED patients to test concentrations of inflammation markers, including interleukin 6 (IL-6), chitinase 3-like 1 (CHI3L1), S100 calcium-binding protein B (S100B), lipoprotein-associated phospholipase-A2 (Lp-PLA2), and macrophage migration inhibitory factor (MIF); vascular and cerebrovascular injury-related markers, including intercellular cell adhesion molecule-1 (ICAM-1) and vascular cell adhesion molecule (VCAM-1); and neurodegeneration-associated markers, including alpha-synuclein (α-Syn) and β-secretase 1 (BACE1). Binary logistic regression analysis was performed to analyze the relationship between biomarkers and ED, and receiver operating characteristic (ROC) curves were used to analyze the diagnostic value of biomarkers.

**Results**: A total of 104 patients were included in this study, with an average age of 63.69 ± 7.21. IL-6 (OR = 2.73, 95% CI: 1.66–6.44, *P* = 0.022), S100B (OR = 4.74, 95% CI: 1.88–11.95, *P* = 0.001), and BACE1 (OR = 6.54, 95% CI: 2.57–16.65, *P* < 0.000) were independent biological indicators for the occurrence of ED.CHI3L1, Lp-PLA2, MIF, ICAM-1, VCAM-1, and α-Syn were unrelated to ED. Plasma BACE1 level had a possible diagnostic value for ED [area under curve (AUC) = 0.75, 95% CI: 0.66–0.85], whereas plasma IL-6 (AUC = 0.62, 95% CI: 0.51–0.73) and S100B (AUC = 0.65, 95% CI: 0.54–0.76) levels had little diagnostic value for distinguishing ED vs. non-ED.

**Conclusion**: Higher levels of systemic inflammation marker IL-6, cerebral inflammation marker S100B, and neurodegeneration-associated marker BACE1 are related to ED. Plasma BACE1 may be a potential diagnostic biomarker for ED.

## Background

Delirium, a common acute fluctuating mental state, mainly manifests as a fluctuating state of consciousness, distractibility, loss of orientation, and confusion (Inouye et al., 2014). Depending on the time of onset, delirium following surgery may be emergence delirium (ED) or postoperative delirium (POD). ED refers to an altered mental state during the recovery stage from anesthesia, which is usually transient and is closely related to the type of anesthesia, duration of surgery, and each patient’s underlying condition (Scott and Gold, 2006). Studies show that ED occurs in 6 to 80% of patients and often results in adverse events such as a prolonged stay in post-anesthesia care units (PACUs), patient self-removal of the endotracheal tube, and drainage tube (Lepousé et al., 2006; Scott and Gold, 2006). Current data show that ED and POD are closely related (Sharma et al., 2005; Schenning and Deiner, 2015).

To diagnose delirium, the gold standard is a bedside comprehensive neuropsychiatric assessment by an experienced specialist according to the five criteria specified in the fifth edition of the Diagnostic and Statistical Manual of Mental Disorders (DSM-V). General practitioners may use widely accepted delirium screening tools, including the Confusion Assessment Method (CAM) scale and the Confusion Assessment Method for the Intensive Care Unit (CAM-ICU) scale (Ely et al., 2001; Inouye et al., 2014). Currently, ED is mainly diagnosed based on subjective scale assessments without objective, specific laboratory indicators. Therefore, it is of great importance to find ED-related biomarkers for early diagnosis, which could contribute to outcome predictions and improve patient prognosis.

The mechanism of delirium is unknown, but a growing body of evidence suggests that activation of a neuroinflammatory response, including the activation of systemic inflammation and the activation of inflammation in the central nervous system, is one of the causes of acute cognitive impairment (Subramaniyan and Terrando, 2019). Beingtraumatic stress, surgery activates the immune system, enhances the secretion of inflammatory mediators such as cytokines and chemokines, and activates immune cells to induce a systemic inflammatory response. Next, the integrity of the blood-brain barrier is compromised, and inflammatory mediators and peripheral immunoreactive cells activate microglia and astrocytes, triggering an inflammatory response in the central nervous system and leading to cognitive impairment (Subramaniyan and Terrando, 2019). Studies have reported that the occurrence and duration of delirium following surgery are related to changes in biomarkers related to inflammatory responses and nerve damage in critical care and elderly patients (McNeil et al., 2019).

In this study, we assessed ED with clinical scales and collected plasma samples to test and screen ED-related biomarkers, such as inflammation markers, including interleukin 6 (IL-6; Sun et al., 2017), chitinase 3-like 1 (CHI3L1; Zhang et al., 2018), S100 calcium-binding protein B (S100B; Hall et al., 2013), lipoprotein-associated phospholipase-A2 (Lp-PLA2; Pokharel et al., 2019), and macrophage migration inhibitory factor (MIF; Oikonomidi et al., 2017); vascular and cerebrovascular injury-related markers, including intercellular cell adhesion molecule-1 (ICAM-1; Janelidze et al., 2018) and vascular cell adhesion molecule (VCAM-1; Janelidze et al., 2018); and neurodegeneration-associated markers, including alpha-synuclein (α-Syn; Fayyad et al., 2019) and β-secretase 1 (BACE1; Shen et al., 2018).

## Materials and Methods

### Subjects

**Figure 1:**
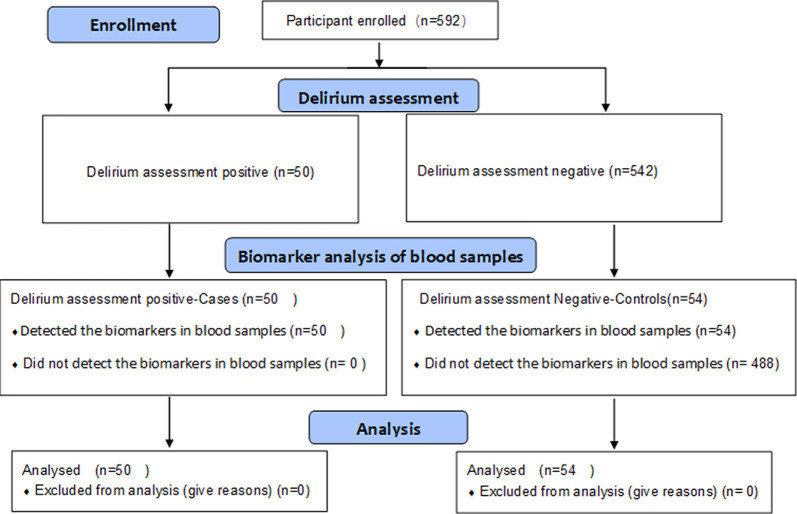
Flow diagram.

Subjects of this study are ED and non-ED patients aged 50 or above who underwent elective laparoscopic surgery under general anesthesia at Xiangya Hospital, Central South University, China, between May 2018 and June 2019. The exclusion criteria were as follows: refusal to participate, inability to cooperate with assessments, preoperative cognitive impairment, and preoperative neuropsychiatric conditions (stroke, epilepsy, Alzheimer’s disease, and schizophrenia). A total of 592 patients were assessed and enrolled for delirium assessment. We analyzed 104 blood samples from ED patients (*n* = 50) and non-ED patients (*n* = 54), respectively (Figure 1). The non-ED patients were selected according to demographic features. Each patient signed an informed consent form before the study. This study was approved by the Ethics Committee of Xiangya Hospital Central South University (201612631) and was registered at the Chinese Clinical Trial Registry (ChiCRT2000031201).

At the end of the surgery, the patients were sent to the post-anesthesia care unit (PACU) and were assessed with the Richmond Agitation Sedation Scale (RASS) and CAM-ICU scale 20 min after the tracheal extubation. The CAM-ICU scale was used if the RASS score was −3 or above (Ely et al., 2003). The CAM-ICU scale assesses the following items:

① acute fluctuations in mental state; ② distractibility; ③ confusion; and ④ altered level of consciousness. ED was diagnosed if the patient presented ① + ② and ③ or ④. Otherwise, CAM-ICU assessment was negative, and non-ED was diagnosed (Ely et al., 2001; Lepousé et al., 2006). If the RASS score was −4 or −5, the assessment was terminated, and the patient was reassessed after the RASS score was −3 or above.

### Sample Processing and Marker Testing

After the assessment, a peripheral blood sample was collected, centrifuged, and then stored at −80°C. An enzyme-linked immunosorbent assay (ELISA) was performed to determine the BACE1 level (DY931/DY008, DuoSet-ELISA Kit, R&D Systems, Minneapolis, MN, USA); and a Luminex assay (Luminex assay kits, R&D Systems, Minneapolis, MN, USA) was performed to determine the plasma level of IL-6, CHI3L1, S100B, Lp-PLA2, MIF, ICAM-1, VCAM-1, and α-Syn. The Concentration of plasma BACE1 was detected by ELISA. Briefly, the plate wells are coated the whole night, then blocked for 1 h. And human plasma was diluted in 0.05% PBST (1:6) and incubated for 4 h. The second antibody, strep-HRP, color reagent AB and stop buffer was added followed the kit instructions. Washing wells with washing buffer were repeated for three times between each step. Quantification of other factors was using Luminex assay kits. Briefly, after centrifugation and vortexing, premixed beads (50 μl well^−1^) were added to 96-well plates. Standards, blanks, and diluted plasma samples (1:1; 50 μl well^−1^) were then added and incubated on a plate shaker (800 rpm) for 2 h. After washing (three times), a biotinylated antibody mixture (50 μl^−1^ well) was added, followed by incubation for 1 h (800 rpm). Repeated washing (three times), the diluted streptavidin-biotin-peroxidase (50 μl^−1^ well) was added, followed by incubation for 30 min (800 rpm). The beads were washed three times and then resuspended in wash buffer. The plate was shaken for 2 min and then analyzed with the Luminex 200 system. All samples were repeated for detection.

### Statistical Analysis

For all biomarkers, the measurements were log-transformed to base-10 to minimize the effect of extreme values. Normally distributed measurement data are expressed as the mean ± standard deviation (*x* ± SD) and were analyzed with the independent sample *t*-test; nonnormally distributed measurement data are expressed as the median (interquartile range) [M (IQR)] and were analyzed with the rank-sum test; count data were analyzed with the chi-square test. We compared the distributions of biomarker values between the ED and non-ED patients using the independent sample *t*-test. Binary logistic regression analysis was individually performed to analyze the independent correlation between each biomarker and ED after adjusting for patient age, American Society of Anesthesiologists (ASA) rating, body mass index (BMI), education level, operation time, and duration of anesthesia. Each biomarker is divided into two categories based on the median to reduce potential interference. For biomarkers with independent correlations with ED, receiver operating characteristic (ROC) curves were used to analyze the diagnostic value of each marker. Since our research focused on the correlation between various laboratory indicators and ED diagnosis, we did not adjust our results for multiple comparisons. SPSS v20.0 was used for statistical analysis (IBM Inc., Armonk, NY, USA).

## Results

### Baseline Data of Patients in the Two Groups

A total of 104 patients were included in the study. Table 1 shows patient age, sex, BMI, ASA rating, preoperative Mini-Mental State Examination (MMSE) score, duration of education, preoperative comorbidities (hypertension, diabetes, and coronary heart disease), smoking history, duration of anesthesia, and duration of surgery. No significant between-group difference was observed in baseline data (*P* > 0.05; Table 1).

**Table 1:** Main characteristics of the study participants.

		Emergence delirium		
Characteristic	Overall (*n* = 104)	Yes (*n* = 50)	Non (*n* = 54)	*P*-value
Age (Y)	63.69 ± 7.21	63.46 ± 8.10	63.91 ± 6.35	0.754
Male sex, *n* (%)	68 (65.4%)	31 (62.00%)	37 (68.51%)	0.623
BMI, (kg/m^2^)	22.46 ± 3.19	21.83 ± 3.16	23.04 ± 3.14	0.053
ASA physical status, *n* (%)				0.771
II	38 (36.54%)	19 (31.00%)	19 (35.19%)	-
III	66 (63.46%)	31 (62.00%)	35 (64.81%)	-
Preoperative MMSE (score)	27.00 (3.0)	27.00 (2.00)	28.00 (3.00)	0.135
Duration of education (Y)	9.00 (6.00)	8.00 (5.50)	9.00 (6.00)	0.292
Smoker, *n* (%)	45 (43.27%)	21 (42.00%)	24 (44.44%)	0.958
Preoperative comorbidities, *n* (%)				
Hypertension	38 (36.54%)	20 (40.00%)	18 (33.33%)	0.616
Diabetes	20 (19.23%)	9 (18.00%)	11 (10.58)	0.954
History of myocardial infarct	5 (4.80%)	4 (8.00%)	1 (1.90%)	0.315
Duration of anesthesia^a^ (min)	267.26 ± 84.24	274.82 ± 85.79	260.26 ± 82.96	0.381
Duration of surgery^b^ (min)	195.28 ± 73.50	200.92 ± 76.23	190.06 ± 71.19	0.454

*^a^Duration of anesthesia: time from anesthesia induction to tracheal extubation; ^b^duration of surgery: time from skin incision to closure; BMI, body mass index; ASA, American Society of Anesthesiologists; MMSE, Mini-Mental State Examination*.

### Plasma Biomarker Levels

No significant between-group difference was observed in the levels of ICAM-1, VCAM-1, MIF, PLA2G7, or α-Syn. For inflammatory markers, the levels of IL-6, chitinase 3-like 1, and S100B were higher in the ED group than in the non-ED group (*P* < 0.05). For neurodegeneration-associated markers, the level of BACE1 was significantly higher in the ED group than in the non-ED group (*P* < 0.05; Table 2).

**Table 2:** The laboratory indexes of patients with or without ED.

Variables	ED (*n* = 50)	Non-ED (*n* = 54)	*P*-value
Log IL-6 (pg/ml)	1.67 ± 0.42	1.47 ± 0.51	0.039^a^
Log CHI3L1 (pg/ml)	4.81 ± 0.47	4.61 ± 0.50	0.041^a^
Log S100B (pg/ml)	2.59 (0.64)	2.09 ± 0.52	0.008^a^
Log Lp-PLA2 (pg/ml)	5.07 ± 0.21	5.04 ± 0.25	0.445
Log MIF (pg/ml)	3.68 ± 0.38	3.60 ± 0.29	0.217
Log ICAM1 (pg/ml)	5.41 ± 0.30	5.44 ± 0.37	0.661
Log VACM1 (pg/ml)	6.07 ± 0.19	6.04 ± 0.26	0.529
Log α-Syn (pg/ml)	2.61 ± 0.11	2.64 ± 0.12	0.222
Log BACE1 (μg/ml)	1.16 ± 0.17	1.01 ± 0.14	<0.000^a^

*^a^*P* value < 0.05; IL-6, interleukin 6; CHI3L1, chitinase 3-like 1; Lp-PLA2, lipoprotein-associated phospholipase-A2; MIF, macrophage migration inhibitory factor; ICAM-1, intercellular cell adhesion molecule-1; VCAM-1, vascular cell adhesion molecule; α-Syn, alpha-Synuclein; BACE1, β-secretase 1*.

### Relationship Between Plasma Biomarkers and ED

Binary logistics regression analysis of potential risk factors showed that after adjusting for age, ASA rating, BMI, education level, operation time, and duration of anesthesia, among the inflammatory markers, IL-6 (OR = 2.73, 95% CI: 1.66–6.44, *P* = 0.022) and S100B (OR = 4.74, 95% CI: 1.88–11.95, *P* = 0.001) were independent biological indicators for ED; among the neurodegeneration-associated markers, BACE1 was an independent biological indicator for ED (OR = 6.54, 95% CI: 2.57–16.65, *P* < 0.000; Supplementary Table 1).

### The Diagnostic Value of Plasma IL-6, S100B, and BACE1 for ED

**Figure 2:**
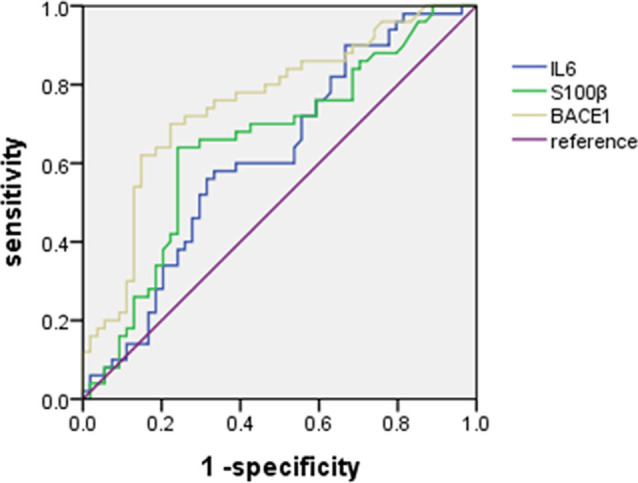
Receiver operating characteristic (ROC) curve for the diagnostic value of plasma IL-6, S100B, and BACEl for ED.

We plotted ROC curves to analyze the diagnostic value of plasma IL-6, S100B, and BACEl for ED (Figure 2). The results showed that plasma IL-6 (Sensitivity = 58.00%, Specificity = 66.67%, AUC = 0.62, 95% CI: 0.51–0.73) and S100B (Sensitivity = 64.00%, Specificity = 75.93%, AUC = 0.65, 95% CI: 0.54–0.76) had little diagnostic value for distinguishing ED with non-ED, whereas plasma BACE1 had possible diagnostic value for distinguishing ED with non-ED (Sensitivity = 70.00%, Specificity = 77.78%, AUC = 0.75, 95% CI: 0.66–0.85; Table 3).

**Table 3:** Diagnostic capability of IL-6, S100B, and BACE1.

Biomarker	Optimal cut-off value	Se (%)	Sp (%)	PPV (%)	NPV (%)	AUC	95% CI	*P*-value
IL-6 (pg/ml)	40.05	58.00	66.67	61.70	63.16	0.62	0.51–0.73	0.041^a^
S100B (pg/ml)	263.68	64.00	75.93	71.11	69.49	0.65	0.54–0.76	0.008^a^
BACE1 (μg/ml)	12.68	70.00	77.78	74.47	73.68	0.75	0.66–0.85	<0.000^a^

*^a^*P* value < 0.05; Se, sensitivity; Sp. specificity; PPV, positive predictive value; NPV, negative predictive value AUC, area under the curve; CI, confidence interval; IL-6, interleukin-6, BACE1, β-secretase 1*.

## Discussion

This study showed that plasma IL-6, S100B, and BACE1 were independent biological indicators for ED. Inflammation markers CHI3L1, Lp-PLA2, and MIF, vascular and cerebrovascular injury-related markers ICAM-1 and VCAM-1, and neurodegeneration-associated marker α-Syn were not independent biological indicators for ED.

Our study found that the BACE1 level was significantly higher in ED patients than in non-ED patients and the BACE1 level was a potential biological indicator with possible diagnostic value for ED. However, there was no direct research showing that BACE1 is associated with delirium currently. BACE1 is a rate-limiting enzyme for the production of β-amyloid protein (Aβ). Most studies on BACE1 focused on Alzheimer’s disease (Vassar, 2014; Yan and Vassar, 2014). A study showed that the BACE1 level is related to mild cognitive impairment (MCI) due to Alzheimer’s disease (Alexopoulos et al., 2018; Shen et al., 2018). A cross-sectional study reported that increased plasm BACE1 level was associated with MCI in type two diabetes (Tian et al., 2020). The growing evidence shows that neuroinflammation makes an important impact on the pathophysiology of delirium (Alam et al., 2018). Nuclear factor-kappa B(NF-κB) is implicated in inflammation, apoptosis, and the transcription of BACE1, which enhances Aβ generation (Sambamurti et al., 2004; Chen et al., 2012). Some studies showed that some compounds could mitigate amyloidogenesis and cognitive impairment *via* inhibiting the NFKB signal pathway to reduce the activity of BACE1 (Choi et al., 2012; Satomoto et al., 2018). These results indicated that BACE1 may be related to ED associated with a neuroinflammatory response, and ED may be a preclinical phase of Aβ related cognitive impairment.

In critical care patients, plasma IL-6 is related to the severity and duration of delirium, suggesting that systemic inflammation is involved in the occurrence and development of delirium (McNeil et al., 2019). This is consistent with the results of this study showing that the IL-6 level was significantly higher in ED patients than in non-ED patients and that IL-6 level was an independent biological indicator for ED, suggesting that a systemic inflammatory response was relevant to ED. However, as a systemic inflammatory marker, IL-6 has little diagnostic value for ED. S100B is secreted by astrocytes and is a marker of cerebral inflammation; its expression level is related to cognitive changes associated with various diseases (Baptista et al., 2017; Lapa et al., 2017). However, its correlation with delirium is inconclusive. In critical care patients, no significant difference of S100B level was observed between delirium patients and non-delirium ones (McNeil et al., 2019); nevertheless, S100B level was significantly associated with delirium in elderly patients with hip fracture surgery (van Munster et al., 2010). Although our study showed that the S100B level was independently correlated with ED, further research is still needed to confirm its diagnostic value for ED.

Our study indicated that there was no significant difference in other inflammatory markers, such as MIF and PLA2G7, between ED patients and non-ED ones. Due to the complexity of the systemic inflammatory responses, diverse inflammatory molecules are produced at different times. We surmise our result might be related to the fact that these inflammatory markers were not produced or already decomposed at the time of detection. ICAM-1 and VCAM-1 are cytokines produced by endothelial cells during vascular wall injury. They enable various inflammatory cells to adhere and infiltrate around brain tissue, thereby amplifying the inflammatory response (Seth et al., 1991; Janelidze et al., 2018). Their levels are significantly elevated in critical care patients but not in ED patients. Also, we analyzed plasma CHI3L1 levels. Some studies have shown that elevated CHI3L1 in cerebrospinal fluid may predict Alzheimer’s disease (Abu-Rumeileh et al., 2019) and that CHI3L1 is also an inflammatory biomarker for multiple sclerosis (Gil-Perotin et al., 2019). In our study, the CHI3L1 level was higher in ED patients than in non-ED patients; however, CHI3L1 was unrelated to ED after adjusting for risk factors, which may be due to different pathological and physiological processes of delirium, Alzheimer’s disease, and multiple sclerosis. Moreover, it should be noted that we analyzed CHI3LI levels in peripheral blood samples, not cerebrospinal fluid. As a marker of neurodegeneration-associated, α-Syn can be detected in cerebrospinal fluid and peripheral blood. In many studies, serum and plasma α-Syn levels have been used as candidate biomarkers for Parkinson’s disease and Lewy body-related dementia, but researchers are still debating whether there is a significant difference in total α-Syn in blood between these patients and healthy subjects (Irwin and Hurtig, 2018). Some studies show that the α-Syn level in the myenteric plexus is an effective biological indicator for POD after gastrointestinal surgery (Sunwoo et al., 2013); this study showed that there was no significant difference in plasma α-Syn levels between ED patients and non-ED ones and the plasma α-Syn level was unrelated to ED. This may be related to variation in α-Syn levels between plasma, cerebrospinal fluid, and the myenteric plexus; plasma α-Syn levels cannot predict ED.

In summary, plasma IL-6, S100B, and BACE1 are related to ED. ROC curves show that plasma BACE1 levels may be a potential biomarker for ED diagnosis and prediction. However, in this study, we only analyzed biomarkers in blood samples, not cerebrospinal fluid. Also, we only analyzed biomarkers after the onset of ED and did not investigate the evolving trends of these biomarkers. This study shows that some inflammatory markers and neurodegeneration-associated markers are related to ED, but we are unable to evaluate whether these markers can predict ED. In the future, further research is needed to analyze changes in biomarkers before (baseline), during, and after delirium, to further clarify the relationship between these markers and ED.

## Conclusion

In this study of patients undergoing elective laparoscopic surgery under general anesthesia, higher levels of systemic inflammation marker IL-6, cerebral inflammation marker S100B, and neurodegeneration-associated marker BACE1 are related to ED. Plasma BACE1 may be a potential diagnostic biomarker for ED.

## Data Availability Statement

The original contributions presented in the study are included in the article/Supplementary Material, further inquiries can be directed to the corresponding author.

## Ethics Statement

The studies involving human participants were reviewed and approved by Ethics Committee of Xiangya Hospital Central South University (201612631). The patients/participants provided their written informed consent to participate in this study.

## Author Contributions

EW and FG: study design. CY, YZ, SL, and YC: patient recruitment and data collection. CY and YC: drafting of the manuscript. CY, YZ, and SL: statistical analysis. FG: technical and material support. EW: critical revision. All authors contributed to the article and approved the submitted version.

## Conflict of Interest

The authors declare that the research was conducted in the absence of any commercial or financial relationships that could be construed as a potential conflict of interest.
