# Supplementary material for: Correlation of Serum BACE1 With Emergence Delirium in Postoperative Patients: A Preliminary Study
Source: Front Aging Neurosci. 2020 Oct 28;12:555594. doi: 10.3389/fnagi.2020.555594 (PMC7655534; doi:10.3389/fnagi.2020.555594)
Supplement: Supplementary file 1 [file Table_1.DOCX]

**Table S1 Associations of laboratory indexes with ED**

| Biomarker | Covariates | OR (95% CI) | P value |
| --- | --- | --- | --- |
| Log IL-6 |  | 2.73 (1.16, 6.44) | 0.022^a^ |
| Log CHI3L1 |  | 1.77 (0.77, 4.06) | 0.180 |
| Log S100B |  | 4.74 (1.88, 11.95) | 0.001^a^ |
| Log Lp-PLA2 |  | 1.35 (0.61, 3.01) | 0.461 |
| Log MIF |  | 1.49 (0.66, 3.36) | 0.339 |
| Log ICAM-1 |  | 0.68 (0.30, 1.55) | 0.357 |
| Log VACM-1 |  | 1.03 (0.45, 2.36) | 0.937 |
| Log α-Syn |  | 1.31 (0.58, 2.98) | 0.522 |
| Log BACE1 |  | 6.54 (2.57, 16.65) | <0.000a |
|  | Age | 0.69(0.25,1.90) | 0.476 |
|  | BMI |  | 0.059 |
|  | BMI(1)^A^ | 0.79(0.05,13.37) | 0.871 |
|  | BMI(2)^B^ | 0.45(0.03,7.65) | 0..577 |
|  | Duration of education | 0.59(0.22,1.58) | 0.296 |
|  | ASA physical status | 0.77(0.26,2.29) | 0.640 |
|  | Duration of anesthesiaa (min) | 1.00(0.99,1.02) | 0.652 |
|  | Duration of surgeryb (min) | 1.00(0.98,1.02) | 0.933 |

^a^P value<0.05

CI, confidence interval

IL-6, interleukin 6; CHI3L1, chitinase 3-like 1; Lp-PLA2, lipoprotein-associated phospholipase-A2; MIF, macrophage migration inhibitory factor; ICAM-1, intercellular cell adhesion molecule-1; VCAM-1, vascular cell adhesion molecule; α-Syn, alpha-Synuclein; BACE1, β-secretase.

BMI(1)^A^:24≤BMI＜28；BMI(2)^B^≥28
